# Public Men’s Clinic: Men’s experiences of healthcare professionals and environment

**DOI:** 10.4102/phcfm.v17i1.5153

**Published:** 2025-11-08

**Authors:** Lawrence L. Mamabolo, Isaac Sibiya, Fezile Buthelezi

**Affiliations:** 1 Community Psychosocial Research (COMPRES), North-West University, Gauteng, South Africa; 2 Anova Health Institute, Sedibeng, South Africa; 3 Anova Health Institute, Johannesburg, South Africa

**Keywords:** men’s health, Public Men’s Clinic, health-seeking behaviour, qualitative research, phenomenology, public healthcare environment

## Abstract

**Background:**

Men often face social barriers linked to norms and systemic issues when engaging with public healthcare services. South Africa’s first Public Men’s Clinic (PMC) was established in 2020, and now, 20 more clinics operate across the country within traditional government clinics. Staffed mainly by male healthcare providers, they are tailored to address men’s health needs, but no published scholarly studies have yet reported on their environment or effectiveness in South Africa.

**Aim:**

The authors aimed to investigate men’s experiences of healthcare professionals and the clinical environment at a South African PMC.

**Setting:**

The study setting was a peri-urban PMC in a community health centre (CHC) in Sedibeng District, Evaton.

**Methods:**

This qualitative descriptive phenomenological study collected data from 43 men through four in-person focus group discussions (FGDs). The findings were thematically analysed.

**Results:**

Participants reported two themes from the FGD: (1) negative healthcare experiences at previous traditional clinics they had attended and (2) positive healthcare experiences at the PMC. Despite general challenges faced in the past at public healthcare facilities, they overwhelmingly reported improvement at the PMC.

**Conclusion:**

Its conducive environment and helpful personnel made participants more comfortable and willing than before to engage with healthcare services.

**Contribution:**

This study, the first scholarly study of men’s experiences of a South African PMC, offers a promising point of departure for broader, more wide-ranging investigations and a benchmark for service providers wishing to set up and run PMCs in their own facilities.

## Introduction

In South Africa, men experience a disproportionately high burden of both fatal and non-fatal diseases, dying prematurely from largely preventable and manageable conditions such as human immunodeficiency virus (HIV) and a range of non-communicable diseases.^1,2^ Men are also less likely than women to seek health services, and when they do, they tend to be very sick.^2,3^ Common barriers to attend healthcare facilities include long waiting times, dissatisfaction with healthcare providers, limited information and concerns about privacy and confidentiality.^3^ Furthermore, South Africa’s National Strategic Plan on HIV, tuberculosis (TB) and sexually transmitted infections (STIs) notes that when men do access services, they are often not provided with comprehensive care such as HIV testing or wellness screenings.^4^ These barriers hinder progress towards national targets such as those in the National Development Plan (NDP)^5^ and the South African National Integrated Men’s Health Strategy 2020–2025.^6^ The NDP, which aligns 74% with the sustainable developmental goals (SDG),^7^ emphasises inclusion and health equity. In response, the Men’s Health Strategy promotes gender-informed approaches, stakeholder collaboration and integration of men’s health into primary care services to reduce male mortality and improve their well-being.^6^

Public Men’s Clinics (PMCs) represent a groundbreaking approach aimed at motivating men to participate actively in the public health system, fostering a culture of proactive health-seeking behaviour (HSB) to enhance overall well-being.^8^ This pioneering initiative was born out of recognition of the challenges that men commonly face as outlined above.^1^ Public Men’s Clinics are clinics located within existing government traditional clinics; they offer a one-stop centre where men receive services for acute and chronic illnesses, most often from a male service provider (a doctor, nurse or counsellor).^8^

The concept of PMCs was first introduced in Bangladesh in 1995 to address the high morbidity and mortality of STIs among men; the initiative proved effective enough to become a permanent fixture offering broader men’s health services.^9^ A similar model was implemented in Kenya between November 2014 and November 2015, where male service providers were employed in a male-only section of a public clinic, resulting in increased HSB and return visits among men.^8^ South Africa’s first PMC was launched in 2020 at Karl Bremer Hospital in Cape Town by the Western Cape government to normalise men’s access to healthcare.^10^ Thereafter, by 2024, 20 PMCs were established across the country, supported by the Anova Health Institute (a non-governmental organisation [NGO] supporting the National Department of Health with technical support) in partnership with the Department of Health districts of Limpopo, Gauteng and the Western Cape. There are fourteen PMCs in Gauteng, one PMC in Cape Town and five PMCs in Limpopo (personal communication, Anova Health National Men’s Health Technical Support Specialist; Beloved Manasidze, July 2024). Following their establishment, however, we could find no published scholarly study that explored their effectiveness or workings. To fill this knowledge gap, our study set out to determine their effectiveness by exploring the experiences of a cohort of men attending one PMC.

By establishing accessible and specialised clinics tailored to men’s unique health needs such as support systems that have a potential influence on health-seeking in men, addressing embarrassment of being diagnosed with chronic diseases such as diabetes, dealing anxiety about their perception of ‘masculine’ if seen ailing and issues with communication regarding their health concerns, we will positively address some of men’s health needs.^11^ Public Men’s Clinics seek to create a bridge between healthcare-seeking behaviour and the male population. It would be valuable to hear men’s experiences about the services rendered at PMCs and the effects of such services on their HSB, to understand the status quo and plan informed interventions, hence this qualitative phenomenological study. The imperative to implement PMC initiatives aligns with the South African national government’s commitment to fulfil the mandates outlined in the NDP 2030^5^ and The South African National Integrated Men’s Health Strategy 2020–2025.^6^ These PMCs not only signify a commitment to individual health but also play a crucial role in realising broader societal goals embedded in the South African National Department of Health vision for comprehensive healthcare.

This study aimed to explore men’s experiences of healthcare professionals and the physical environment within a particular PMC. Understanding these experiences is important because they shape men’s willingness to seek and continue care. By identifying barriers and positive experiences men face within the public health system, the study can inform strategies to create more welcoming, male-friendly health services.

## Research methods and design

This study employed a qualitative, descriptive, phenomenological design to explore and understand the lived experiences of men attending the selected PMC. The descriptive tradition of phenomenology originated from the writings of Husserl, which were further developed by Merleau–Ponty.^12^ This method of inquiry emphasises the lifeworld of participants as a starting point as it is focused on the lived experience of participants, in this instance, men attending PMC.^13^ Furthermore, the philosophy of phenomenology is the study of a phenomenon that is something as it is experienced (or lived) by a human being, which means how things appear in their experiences of attending PMC.^14,15,16,17^

We conducted the study at Levai Mbatha Community Health Centre, located in Evaton, in the Emfuleni Local Municipality, and it is one of the two facilities offering PMC services in the Sedibeng District. This site was selected for its functionality, sufficient resourcing and status as a prospective Ideal Community Health Centre (CHC).^18^ Men attending the PMC were redirected from the main clinic to the PMC where services were rendered exclusively by male staff (nurses, doctors, counsellors).

A homogeneous purposive sampling approach was used to recruit participants who shared key characteristics, notably gender (all were men) and attendance at the PMC. Etikan et al.^19^ stated that participants in homogenous sampling would be similar in terms of ages, cultures, jobs or life experiences. The idea is to focus on this precise similarity (men and experiences at PMC) and how it relates to the topic being researched.^20^

A total of 43 male participants took part in four focus group discussions (FGDs) held in September 2024 and October 2024. The groups were composed of 12, 12, 10 and 9 participants, respectively. Inclusion criteria required participants to be over 18 years old, have prior experience attending a mainstream clinic and have attended the PMC at least once, with a follow-up appointment within a month to reduce inconvenience. Clients not meeting these criteria or who refused their consent were excluded.

Clinicians and counsellors screened clients for our study, using the inclusion criteria during their regular consultations. Eligible and interested participants were referred to the on-site researcher who was present during recruitment days in August 2024 and September 2024. The researcher explained the study, provided a pamphlet and consent form, and recorded participants’ contact details. Participants received reminder calls a week before their follow-up visit. On the appointment day, after their clinical consultations, participants were directed to the Levai Mbatha boardroom, a comfortable and private space. They signed consent forms facilitated by an independent person who was a male counsellor working at another clinic. They then took part in FGDs facilitated by I.S., with L.L.M. acting as observer, following the recommendation by Clark et al.^21^ Sessions began with participants setting ground rules. Debriefing took place with the observer after the session, after which each participant received R50.00 according to the Time Inconvenience and Effort (TIE) principle,^22^ followed by refreshments.

As the study sought to investigate men’s lived experiences of attending the PMC, guided by the overall research aim, the FGDs explored six key questions given as follows: (1) *What are the barriers that hinder men from attending clinics?* (2) *What are your past experiences of clinic attendance?* (3) *What are your experiences of the services offered at the PMC?* (4) *What is the impact on your overall health of attending the PMC?* (5) *How does peer interaction and receiving care from male service providers affect your experience?* and (6) *What motivates you to seek healthcare services at the PMC?* These questions aimed to address South Africa’s current knowledge gap on men’s health and how tailored intervention such as PMC is experienced by men.

Saturation was reached by the third FGD. As we were reviewing data collected on a regular basis, little or no new information on the research topic emerged.^23,24,25^ Focus group recordings were transcribed and translated into English. All researchers are fluent in English, Southern Sotho and IsiZulu, which were languages used interchangeably during discussions. The data were analysed using Braun et al.’s six-phase thematic analysis framework, that is, familiarisation, coding, theme generation, reviewing, defining and naming themes, and report writing.^26^ The first phase was to familiarise ourselves with the data. Before we begin evaluating specific items, it is critical to acquire a complete overview of all the data we have gathered. From the FGDs that were performed, we took all the notes, and audio recording that needed to be transcribed, and read through the texts and took notes, in order to be familiar with the data. This entailed reading the data over again to find any patterns or meanings and make notes to be able to jot down potential codes. For transcribing the audio from the interviews conducted, we used verbatim transcription (after translations if necessary), which captured every detail from the audio file, in order to not miss any important message that was given.^27^

The second phase, which was coding the data, involved creating meaning. It is a process of making specific text passages that could be a sentence or a phrase and assigning brief labels to them that better convey meaning.^26^ This involved using the line-by-line coding, as we have to develop a new set of code, as we used an inductive analysis. Atlas.ti helped in coding, which possesses resources for storing and retrieving encrypted information, or alternatively, the use of a table in a word document was the most relevant for this research study, which was used to code the data.^28^

The third phase, which was creating themes, demands that codes and data be interpreted actively, whereas with codes, they just indicate important facts.^29^ The first step involves reviewing the list of codes and the extracts that goes along with them and grouping the codes into more general themes that reveal something intriguing about the data. During this process, we used visuals, mind maps or tables, which can help in getting a thorough understanding. Data trends and patterns are reflected by themes and sub-themes, which can be created by combining several codes.^26^ The use of networks as a way to organise theme was used. Networks are made up of blocks or nodes that connect themes. Themes and sub-themes were created, and each is capable of showing various types of data.^23^

In the fourth phase, we reviewed potential themes. For this step, it is imperative for the researchers to go back and check which element we could have missed, implying that some labels might better fit into other themes than their original. We went back to the data set, and contrasted our themes with it, and asked questions on whether something is missing, or does the data actually support the themes, and if not, can it be altered to improve the effectiveness of the themes.^26,27^

In the fifth phase, we defined and named themes. This is a crucial step in data analysis; therefore, the selection of labels of themes that are ‘catchy’ and convey the analysis’s main ideas are a great step to go about.^26^ The conciseness, relevance and consistency, as well as the explanatory value, of each label are all checked, and therefore, if necessary, titles are modified, clarified, removed and merged to better the labels.

Lastly, we wrote the report on the findings, which is the last step of thematic analysis.^25,26^ When writing a report, it is crucial that you provide detailed information, in order for the person reading to be able to understand and comprehend the analysis, meaning that the reader should be able to ask questions like ‘why’ or ‘when did you investigate’ and still be able to get the answer from the data analysed. This means that the data collected and the conclusions drawn from the data should be concise and well understood, that is, within and across the themes.^26^

### Trustworthiness

Creditability in this study was checked through member checking, that is, by going back to the participants after the data were analysed to check that the data analysed were true to the participants’ intentions. Participants were contacted through their preferred method of contact on the consent form to schedule an appointment to review the analysed data. Data analysis software (Atlas.ti) was also used.^28^ Because one of the researchers is the co-ordinator of men’s health, there is a potential risk of recruitment bias. Men who have had positive experiences with the clinic may feel more willing to participate, while those with negative experiences may decline, resulting in an overrepresentation of favourable views.^27^ There is also a possibility that participants may feel pressured to participate or respond in socially desirable ways to please the researcher.^25^ To mitigate this bias, recruitment was done through neutral clinic staff who distributed invitations, and if participants agreed, they will contact the other researchers who were available on recruitment days. Furthermore, participation was entirely voluntary. We further requested colleagues who can analyse qualitative data and check our audit trail to assist in analysing the data to compare our conclusion with their conclusion. To further enhance dependability, we used the audit trail, that is to have process logs or journal, in which we kept track of all decisions, and it was also useful in data analysis to explain how we reached certain conclusions.^26^ For transferability, we strived to provide rich and detailed descriptions of our findings, to enable readers to understand the nuances and complexities of the phenomenon, men and their PMC engagement experiences, which was being studied.^23^

Reflexivity and bracketing were important in maintaining objectivity, as two of the researchers worked in the men’s HIV prevention sector. Bracketing minimised the influence of personal biases, ensuring that data interpretation was based on participants’ narratives.^12^ This enhanced both the objectivity and validity of the study.^11^ Reflexivity was maintained by the researchers keeping a journal to critically reflect on their dual roles, personal assumptions and potential influence on participants’ responses throughout data collection and analysis.^11,13^ These steps enhanced the credibility and trustworthiness of the findings.

### Ethical considerations

Ethical approval to proceed with the study was granted by the North-West University Health Research Ethics Committee (NWU-HREC) with reference number NWU-00084-24-A1 and Sedibeng Health Research and Ethics Committee with reference number GP_202401_028. Another issue to consider in FGDs is the fact that full confidentiality cannot be upheld as we might not have control on what other participants in the group might say after discussions. To curb this, we also included a partial confidentiality clause in the consent form,^25^ and this was discussed before the FGDs could commence. Participants were requested to sign the consent form on the day of the FGD before the commencement of the interview.^21^ Anova Health Male Counsellor working at a different facility assisted on the day. He explained the consent form again individually to participants in their preferred language and gave them an opportunity to ask questions before they were requested to sign the consent form. Participants were informed verbally and in the written consent form that the FGDs will be recorded. Data security protocols were followed. All recordings were deleted from devices post-transcription and stored in a password-protected cloud, while physical documents were kept under lock and key.

## Results

A total of 43 male participants participated in four FGDs. The first focus group consisted of 12 participants with an average age of 45 years; the youngest participant was aged 28 years, and the oldest participant was aged 72 years. Four participants from this group reported being unemployed.

**TABLE 1:** Themes and sub-themes.

Themes	Sub-themes
1. Negative experiences at a previous traditional clinic	1.1. Long waiting times 1.2. Disrespect from health practitioners and other staff members
2. Positive experiences at the PMC	2.1. Efficiency and professionalism 2.2. Satisfaction with men-to-men services 2.3. Encouragement from health practitioners (empowerment) 2.4. Male support bonding in queues 2.5. Recommendations by participants on areas of improvement

*Source*: Dowden J, Mushamiri I, McFeely E, Apat D, Sacks J, Ben-Amor Y. The impact of ‘male clinics’ on health-seeking behaviors of adult men in rural Kenya. PLoS One. 2019;14(11):e0224749. https://doi.org/10.1371/journal.pone.0224749

Note: Traditional clinic: normal clinic where genders go together for treatment of ailments; Public Men Clinic: dedicated clinic for men for treatment of acute and chronic illnesses.

PMC, public men’s clinic.

The second focus group had 12 participants with an average age of 39 years; the youngest participant was 24 years old and the oldest participant was 71 years. Seven participants reported being unemployed in the second focus group.

The third focus group consisted of 10 participants with an average age of 46 years, of whom the youngest participant was 21 years old, and the oldest participant was 75 years old. Seven participants reported being unemployed in the third focus group.

The fourth focus group comprised nine participants, with an average age of 51 years; the youngest participant was 29 years old, and the oldest participant was 72 years old. Seven participants in the fourth focus group reported being unemployed.

### Theme 1: Negative experiences at a previous traditional clinic

This theme captures everyday challenges that the participants routinely experienced at traditional public health clinics. The sub-themes reveal systemic issues that contributed to negative patient experiences, reduced trust in healthcare services and had the potential to discourage HSB. Participants commonly reported long waiting times, breaches in confidentiality and disrespectful treatment by staff. These issues are not exclusive incidents but rather form a regular part of the daily experiences of many individuals in our study who were seeking care in public healthcare facilities.

#### Sub-theme 1.1: Long waiting times

One of the most commonly cited frustrations in our sample was the excessive time patients had spent waiting for services. Participants described arriving at clinics early only to be seen hours later:

‘Tuesday I was here I stayed at the main clinic site from 12H00 and then I was not yet assisted by 15H00. I asked where the people are then they said they are on lunch then I remembered that my stupidity I came to the main clinic instead of the men clinic.’ (Mens FGD Sedibeng Group 3, Participant 3, 57 years old, unemployed)

Another participant complained about how the queue is managed, leading to long waiting times:

‘You know Mr Interviewer we always come back to the same conclusion, you know I spent a lot of time here I have to answer this you know I needed this procedure because I used to come here, the queue is the challenge.’ (Mens FGD Sedibeng Group 2, Participant 2, 31 years old, unemployed)

#### Sub-theme 1.2: Disrespect from health practitioners and other staff members

Participants raised concerns about the way in which their private medical information was handled. In some clinics, consultations took place in open areas where others could overhear sensitive information:

‘Can we please fix this thing of people are saying if you are HIV positive you must sit there if you have diabetes you must sit there so that makes us not to have confidentiality because now people know now what is wrong with us so you can see why men are not coming because these nurses are exposing us they are saying we must sit there and when you sit there people know what is wrong with you, you know.’ (Mens FGD Sedibeng Group 2, Participant 3, 52 years old, unemployed)

Participants shared experiences of being treated rudely or dismissively by healthcare staff. This behaviour fostered feelings of humiliation as well as reluctance to return to the clinic:

‘You know what happened to me when I went there? I broke my leg playing soccer, I was complaining because of pain, the doctor stood me up but by the time he was assisting me he was telling me that I talk too much I should keep quiet. Yeah but it wasn’t me it was the pain speaking too much.’ (Mens FGD Sedibeng Group 2, Participant 1, 38 years, unemployed)

Another participant elaborated angrily: he had stopped taking his chronic medication and discovered that another member of staff who had nothing to do with his clinical team had got to know his clinical condition:

‘There is always something that will happen that will make me angry when I come to the clinic. I can tell you, in the year 2010 they treated me [so] bad that I stopped taking my medication for high blood. From that day I was so angry I left treatment. The people that work there they have attitude, even security.’ (Mens FGD Sedibeng Group 3, Participant 3, 57 years, unemployed)

### Theme 2: Positive experiences at the Public Men’s Clinic

This theme explores men’s direct experiences with the designated men’s health clinic, reflecting both structural and interpersonal aspects of care. Unlike normal traditional clinics where men often feel marginalised, many participants reported more positive experiences in men’s clinics, highlighting improvements in efficiency, professionalism, empowerment and peer support. These PMC facilities seemed to provide a more affirming environment tailored to the unique needs and preferences of male patients, which led to greater satisfaction and engagement.

#### Sub-theme 2.1: Efficiency and professionalism

Participants frequently cited the operational efficiency of men’s clinics as a major factor in their positive experiences. Shorter wait times, streamlined services and clear procedures contributed to a more user-friendly environment:

‘Mr Nurse make things to be a bit easier; you know when you go inside there you don’t have many things that will delay you. You do your vital signs and after you will be assisted. You know Mr Nurse can run the clinic by his own, you know when you can go check if it’s 12:00 in the afternoon, you’ll find the men clinic empty because they work.’ (Mens FGD Sedibeng Group 2, Participant 3, 52 years old, unemployed)

The conduct and competence of healthcare professionals were also praised. Participants described staff at men’s clinics as respectful, attentive and well informed:

‘At men’s clinic, the first thing I saw when I came in is that they take confidentiality of a patient very serious, … because they have made this space for us men it really has made an impact and also its good that you get assisted by a male nurse as well, you are able to talk to him. With me I haven’t received any problem since I have been attending there.’ (Men FGD Sedibeng Group 1, Participant 8, 34 years, employed)

#### Sub-theme 2.2: Satisfaction with men-to-men services

A key highlight of our findings was the participants’ preference for male providers and men-centred services. Many felt more comfortable discussing sensitive issues with male healthcare professionals:

‘[*I*]t’s meant to be man to man when people are men to men, they can speak things that are very sensitive.’ (Mens FGD Sedibeng Group 3, Participant 1, 76 years old, unemployed)

Another participant indicated that he was happy to look forward to being assisted by a male nurse when at a PMC:

‘Guys, I’m happy with this; you see the male nurses inside there are very good so we are man to man so, you know, when I go into that room I know there’s a man waiting to assist me so we speak man to man. So far it’s only good things that I can talk about the men’s clinic, nothing bad that I can see or say.’ (Mens FGD Sedibeng Group 2, Participant 1, 38 years old, unemployed)

Overall satisfaction with the services was high. Participants described the clinical environment as welcoming, organised and emotionally safe, in stark contrast with their experiences at traditional clinics:

‘You know I attended men’s clinic, and these people are very good, they push that line. They are too much better than the main clinic. Guy’s I’m happy with this. You see the male nurses inside there are very good.’ (Mens FGD Sedibeng Group 2, Participant 1, 38 years old, unemployed)

Another participant indicated that he was satisfied with the comprehensive assessment done at the PMC:

‘You know the clinic – I had always thought it’s for people who have problems of manhood. Example, I was always saying that to people, but when I went there oh I was assisted very fast and I was also surprised that they check everything, you see. Blind perception, so we should practise the right message; I also told my friend and then he laughed at me.’ (Mens FGD Sedibeng Group 3, Participant 10, 32 years old, unemployed)

#### Sub-theme 2.3: Encouragement from health practitioners (empowerment)

Participants also appreciated being encouraged by health professionals to take charge of their own well-being. This empowerment included education, motivation and destigmatisation of health conditions:

‘I went to men clinic; I got this male nurse and he tested me everything and give me even advice on how to take care of my health – he even took me to the stage of seeing that our health is being taken serious.’ (Mens FGD Sedibeng Group 2, Participant 2, 31 years old, unemployed)

#### Sub-theme 2.4: Male support bonding in queues

Interestingly, some participants reported positive social interactions while queuing at the PMC and described informal bonding with other men who shared similar experiences:

‘As men we like to hang out with people and talk, and when you go there and you find another man and you talk to him and we end up opening up to each other and we know that when I leave here I have spoken to someone who is like me there and [*we*] advised each other. You won’t find another men going around discussing another men’s issues with other people. That is what is making us.’ (Mens FGD Sedibeng Group 4, Participant 3, 29 years old, unemployed)

Another participant indicated that when he was at the PMC, he felt free to engage with other men as there were only men in the queue, whereas at the normal clinic, this was not the case:

‘Me personally I like men’s clinic, I see people are free; but when they are at the main clinic people are not free and they don’t talk, but when they are at men’s clinic they do talk and they are free.’ (Mens FGD Sedibeng Group 4, Participant 1, 32 years old, employed)

#### Sub-theme 2.5: Recommendations by participants on areas of improvement

Although participants talked about positive experiences at PMC, they also have some recommendations to make the clinic more appealing and address their other needs. One talked about the expansion of the PMC model:

‘[*Y*]eah, you know if they expand these clinics then everyone will be able to go to the clinic closer to where there are. You see I’m from Everton north and I come here, and I pay to come here. I pay taxi.’ (Mens FGD Sedibeng Group 3, Participant 6, 32 years old, employed)

Another indicated that the services should be expanded to open the PMC even on weekends:

‘On the weekends you remember one of our brothers already talked about the weekend the weekend you can’t get help that you came for and there is no separation you know.’ (Mens FGD Sedibeng Group 2, Participant 4, 36 years old, employed)

## Discussion

This qualitative study explored the experiences of 43 men attending a PMC in the Sedibeng District, focusing on their past experiences at traditional public clinics and their recent experiences at the PMC. The findings revealed two dominant themes: (1) negative past experiences at traditional public clinics and (2) more positive experiences at the PMC. These contrasting experiences offer valuable and novel preliminary insights into how this South African clinic structure, provider attitudes and gender-sensitive service delivery can shape men’s healthcare behaviours.

Participants widely described traditional clinics as unwelcoming, inefficient and emotionally unsafe. The most frequently cited frustrations were long waiting times, poor queue management and inconsistent service delivery. These issues mirror systemic challenges documented across South Africa’s public healthcare sector, including staff shortages, overcrowding and operational inefficiencies.^30^ For many men in our study, such delays were more than an inconvenience; they represented a broader pattern of neglect and disregard, reinforcing a perception described in other South African studies that traditional clinics are not designed with men’s needs in mind.^3^ Furthermore, these issues extend beyond logistical failings; they amplify men’s internalised masculinities, where enduring discomfort without complaint aligns with hegemonic ideals of stoicism and self-reliance, potentially deterring timely care-seeking.^31^

Another critical concern arising in our study was the lack of privacy and confidentiality in traditional clinics, particularly when clinical practices made patients’ conditions publicly visible, for instance, through the use of condition-specific queues. Such breaches were experienced not just as administrative oversights but as violations of patients’ rights. This aligns with prior research by Chima^32^ showing that confidentiality is central to men’s willingness to engage with healthcare, especially when dealing with stigmatised conditions such as HIV or mental illness. Our findings further illustrate how these breaches intersect with cultural factors, fostering a cycle of avoidance where men delay care to preserve face, aligning with gendered health-seeking theories that frame help-seeking as emasculation.^31^ However, critically, such reports may reflect recall bias, as participants, now attending the PMC, could retrospectively amplify negatives to justify their shift, raising questions about these experiences across diverse socio-economic contexts.

Many of our participants recalled experiences of disrespect by healthcare workers. Reports of being spoken to harshly, dismissed or humiliated (sometimes even by security guards or non-clinical staff) indicated a breakdown of therapeutic trust. Similar findings have been reported in other South African studies, where masculine identity and perceived provider hostility interact to produce disengagement from care.^31,33^ One participant in our study explicitly reported discontinuing chronic medication after a demeaning encounter, illustrating the tangible consequences on the health of poor patient–provider relations.

In stark contrast, our participants’ experiences at the PMC were overwhelmingly positive. Men described the clinic as efficient, respectful, empowering and designed with their needs in mind.^34^ One of the most valued aspects was operational efficiency, and shorter waiting times, simplified procedures and clear patient flow were repeatedly mentioned. These elements made the clinic feel accessible and purposeful, which aligns with previous research in Kenya and Bangladesh, highlighting how structural organisation affects men’s willingness to attend and return to clinics.^8,9^

Professionalism and respectful treatment at the PMC were also praised. Participants described the staff, particularly male nurses, as competent, approachable and mindful of confidentiality. The preference for male healthcare providers, echoed throughout the four FGDs, underscores the value of gender-concordant care in fostering openness, particularly around sensitive issues. This is consistent with the findings by Chavalala et al.^35^ who found that male patients often communicate more freely with male providers, especially in socio-cultural contexts where masculinity norms discourage vulnerability. However, this positivity warrants critique. While novel in demonstrating the PMC’s role in challenging stigma through male-only spaces, it risks essentialising masculinity by assuming all men prefer segregated environments, potentially marginalising those who do not conform to traditional norms.^33^ Moreover, the enthusiasm for male nurses and gender-concordant care, consistent with Chavalala et al.,^35^ fosters openness on sensitive topics but could inadvertently reinforce homosocial bonds that exclude diverse masculinities, such as those of queer or non-binary men, thus limiting inclusivity.

Furthermore, the PMC in our study was described not just as a place of treatment but as a supportive space where men could be educated, encouraged and empowered to take control of their health. This sense of empowerment articulated by participants, reflects Freirean principles of participatory health promotion, which is a collaborative process where clients critically reflect and take action to transform their own health.^36^ Several participants even noticed changes in their own health-seeking attitudes and a desire to share their experiences with peers, indicating the broader social ripple effects of gender-sensitive care.^8^

Finally, some men highlighted the informal social bonding that occurred while queuing, finding that the male-only environment fostered openness and mutual support. This form of peer engagement is rarely documented in clinic settings, yet it may be a strong factor in sustaining men’s participation in healthcare and is worth examining further in the specific PMC context. This suggestion is supported by both Dowden et al.^8^ in Kenya and Chavalala et al.^35^ who argued that male peer networks are key to reducing stigma and normalising help-seeking, particularly where HIV and chronic diseases are concerned.

Despite these strengths, the PMC is not without negatives, including limited operating hours that exclude working men, necessitating improvements such as weekend access and scalability to other traditional clinics.^37,38^ Critically, expanding without addressing underlying resource inequities could strain systems further. Our findings reinforce the value of tailoring healthcare services to the unique needs of men, even though there are areas of improvement like expanding working hours and opening on weekends, including its possible scale-up to other traditional clinics. As demonstrated in this study, PMCs offer a promising model for improving men’s access to and engagement with the healthcare available. Policymakers and health planners could usefully consider developing PMCs further or incorporating equivalent gender-sensitive strategies into existing public health systems. Key features, including male staffing, service efficiency, patient confidentiality and respectful communication, emerged as critical preferences in our investigation but could be explored further for their potential as central components of effective men’s health delivery.

### Study limitations

Although this study is small in scale, it offers valuable insights into men’s healthcare experiences when engaging with the South African public health system. Because it was conducted exclusively in a peri-urban setting, the findings may differ from PMCs in rural areas or private healthcare users. The small, context-specific sample, suitable for qualitative depth, stopped short of reflecting the full diversity of men’s experiences, such as those of Lesbian, Gay, Bisexual, Transgender and Queer (LGBTQ) individuals or migrants. Furthermore, social desirability bias may have influenced responses. In addition, the data represent a single time-point, without capturing evolving health policies or attitudes.

## Conclusion

This study offers contextualised men’s experiences in a South African public health clinic, in this instance, the PMC. It highlights the structural, interpersonal and gendered dynamics that influence men HSBs and experiences and, for the first time, introduces the specific context of the nation’s PMC initiatives. It leads to promising opportunities for further research to explore long-term outcomes of gender-specific care models on men’s health behaviours in PMCs as well as other facilities, particularly in rural and resource-constrained settings. Comparative studies examining male experiences across both traditional clinic and PMCs could provide deeper insights into scalable best practices, and longer-term research projects to track behaviour change and adherence over time could advance understanding of ways in which relational care dynamics affect health outcomes.

By focusing on men’s interactions within targeted clinic environments, our investigation contributes empirical data to support gender-responsive service delivery models in South Africa, with findings that may resonate with men’s care experiences elsewhere as well. They have the potential to significantly inform public health policy by highlighting the importance of dedicated men’s health services in promoting equity and access. We believe that this first investigation into South African patients’ PMC experiences offers an encouraging starting point for broader studies covering the spectrum of PMCs in the country, as well as for longitudinal research into the evolution and impact of such facilities on men’s health both here and abroad.
